# An update for *Halyomorphahalys* (Stål, 1855) (Hemiptera, Pentatomidae) distribution in Belgium

**DOI:** 10.3897/BDJ.12.e125067

**Published:** 2024-06-19

**Authors:** Grégoire Noël, Arnaud Segers, Joachim Carpentier, Luca Rossini, Emanuele Garone, Frédéric Francis

**Affiliations:** 1 Department of Functional and Evolutionary Entomology, Gembloux Agro–Bio Tech, University of Liège, Passage des Déportés 2, B–5030, Gembloux, Belgium Department of Functional and Evolutionary Entomology, Gembloux Agro–Bio Tech, University of Liège, Passage des Déportés 2, B–5030 Gembloux Belgium; 2 Service d’Automatique et d’Analyse des Systèmes, Université Libre de Bruxelles, Av. F.D. Roosvelt 50, CP 165/55, Brussels, Belgium Service d’Automatique et d’Analyse des Systèmes, Université Libre de Bruxelles, Av. F.D. Roosvelt 50, CP 165/55 Brussels Belgium

**Keywords:** Brown marmorated bug, pentatomid ecology, biological control, insect occurrence

## Abstract

The brown marmorated stink bug, *Halyomorphahalys*, represents an important insect pest and subsequently an important agricultural threat due to its polyphagous feeding habits and adaptability to diverse climates. Native from East Asia, its recent establishment in various regions, including North America and Europe, has led to substantial yield losses and economic impacts, which highlight the need for comprehensive research efforts, based on data occurrence by combining those from expert entomologists and citizen scientists. We reported here 14 new occurrences of this insect pest in the three regions of Belgium. Then, these data were merged with data occurrences from other studies and GBIF datasets of Belgium. The combined dataset showed a peak of presence of *Halyomorphahalys* in October and a dominance of field observations from citizen scientists especially in the nothern part of Belgium, Flanders. Crowd-sourced data have provided valuable insights into the presence and distribution of *Halyomorphahalys* in Belgium. Given the importance of the generated dataset, it could be asserted that this pest is uniformly distributed across the entire country, which necessitates additional research to evaluate its impact on various crops.

## Introduction

The brown marmorated stink bug, *Halyomorphahalys* (Stål, 1855) (Hemiptera, Pentatomidae), is an harmful pest native to East Asia and inadvertently introduced, over the years, in different areas worldwide. During the 1990s, *H.halys* reached Pennsylvania, USA (Hoeboeke and Carter 2003) and after two decades successfully colonised the greatest part of North America. Its spread was responsible for significant yield losses and damage to fruit orchards (mainly soft fruit trees and hazelnuts), maize and soybean crops, ornamental plants and various other food crops (Rice et al. 2014). Its presence in Europe was firstly assessed in Switzerland in 2007 (Wermelinger et al. 2008), from where it progressively expanded across Europe up to the Mediterranean Basin (Hess et al. 2022).

The wide diffusion of this sap-sucking pest is endorsed by its high adaptability to different climates and by an exceptional level of polyphagy. The current literature reports a feeding preference for hundreds of host plants (ca. 300) from multiple botanical families (Lee et al. 2013, Rice et al. 2014). Beyond the detrimental impact on agricultural production, *H.halys* is also an annoying pest for many urban centres close to the most infested areas (Leskey et al. 2012, Kibar et al. 2019), since the overwintering adults usually inhabit human dwellings and infrastructures, occasionally forming massive aggregations (Inkley 2012). This manifold behaviour underscores the complex ecological and economic challenges posed by the invasive *H.halys* species.

In Belgium, the taxonomic inventory revealed the presence of 46 distinct species belonging to the Pentatomidae family (Claerebout et al. 2018), where *H.halys* is the latest observed species. This invasive species was firstly reported in Belgium in 2011 within the Municipality of Soignies (Claerebout et al. 2018), but its current establishment and diffusion are still not clearly defined. The greatest portion of the current information on its spatiotemporal distribution patterns relies on crowd-sourced data aggregation platforms, such as *Observations.be* (https://observations.be/; Vanreusel et al. (2024), Paquet et al. (2024)), where citizens actively contribute to the database by reporting new observations.

It is worth pointing out that, on one hand, only a small part of the identifications are rigorously carried out by insect specialists (insects prepared in collections and identified using dichotomous keys), which is essential for precise taxonomic identification that is free from any potential confusion. On the other hand, many reports are provided with pictures of the specimens, that should be validated by specialists. With this precondition, we can say that the crowd-sourced data are surely a helpful tool for researchers, but it needs support from *ad hoc* monitoring carried out in rigorous ways from research centres (Maistrello et al. 2018). In the specific context of Belgium, *H.halys* is relatively straightforward to identify with a few morphological characteristics (Claerebout et al. 2018), especially compared to a highly similar native bug species *Rhapigasternebulosa* Poda, 1761 (Hemiptera, Pentatomidae) (Rot et al. 2018). Validation of records is significantly facilitated when accompanied by verified photographs. Furthermore, few studies (e.g. Serteyn et al. (2020), Serteyn et al. (2021), Berteloot et al. (2024)) broached the biogeography, ecology and biological control of this species in Belgium. Different research centres and universities in Belgium, as well as qualified technicians, are actively controlling the population dynamics of this pest, but, to date, there are no studies that combine and analyse the data together.

Given the high adaptability and diffusion rate of this species, carrying out a dataset update and identifying the situation in Belgium is fundamental. Accordingly, the goal of this data report is to: *i)* collate the new Belgian occurrences considering both crowd-sourcing data and monitoring carried out by experts and, *ii)* update the spatial and phenological distribution of this invasive pest in Belgium.

## Material and methods

Belgian records of *H.halys* were extracted from the GBIF database on 1 February 2024, using the R package *rgbif* (Chamberlain and Boettiger 2017, Chamberlain et al. 2024) and employing the *occ_data* function to filter exclusively Belgian records. A total of 3495 Belgian records were obtained from the GBIF repository. These records were sourced from various databases: iNaturalist n = 87 (iNaturalist contributors, iNaturalist 2024) and Observations.be n = 3408 (Paquet et al. 2024, Vanreusel et al. 2024). These records were validated by specialists based on live specimen pictures. Subsequently, we merged the occurrences primarily sourced from crowd-sourcing with those documented in the study by Berteloot et al. 2024 (n = 99), along with our newly-acquired occurrences. All the collected insects were prepared, labelled and vouchered in the laboratory collection following the insect preparation instruction of Mouret et al. (2007) and Fagot et al. (2022). The dichotomous keys from Claerebout et al. (2018) were used to identify *H.halys* in laboratory conditions. The maps of *H.halys* spatial distribution were generated using *mapview* R package (Appelhans et al. 2019).

### Used acronyms

- ULB: Université Libre de Bruxelles;
- GBIF: Global Biodiversity Information Facility.

## Results

**Figure 1.**
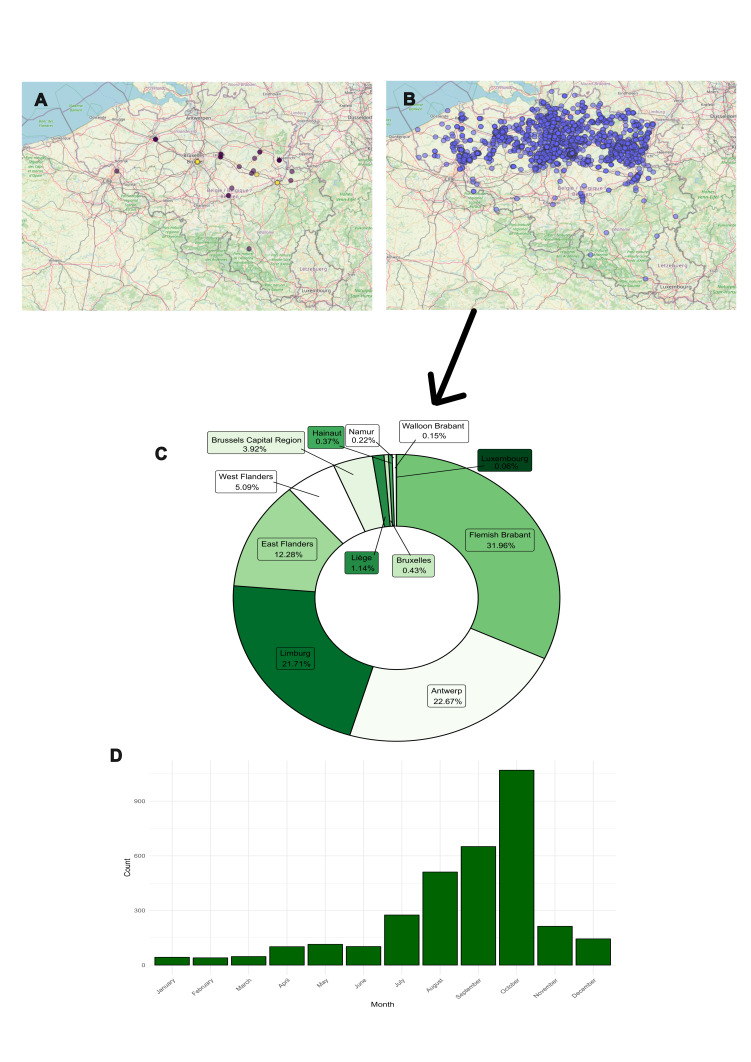
Data distribution of *Halyomorphahalys* (Stål, 1855). **A** New records of *H.halys* (yellow dots) and from Berteloot et al. (2024) study (purple dots); **B** Occurrence of *H.halys* from the GBIF dataset; **C** Spatial distribution of *H.halys* by Belgian provinces of the GBIF occurrences; **D** Phenological distribution of *H.halys* from GBIF occurrences.

In this study, 14 new occurrences of *H.halys* (Fig. 1A) were documented in Wallonia, Flanders and Brussels Regions. After filtering the redundant data, 3172 occurrences of *H.halys* from the GBIF dataset were attributed to Belgium (Fig. 1B). Most of the GBIF’s records are concentrated in the regions of Flanders (93.71%), followed by Brussels-Capital (3.92%) and Wallonia (2.37%), (Fig. 1C). Phenological observations showed the presence of *H.halys* in late summer and early autumn, specifically August, September and October, when the highest count of the year was observed (Fig. 1D).

### New records of H.halys for Belgium

**Figure 2.**
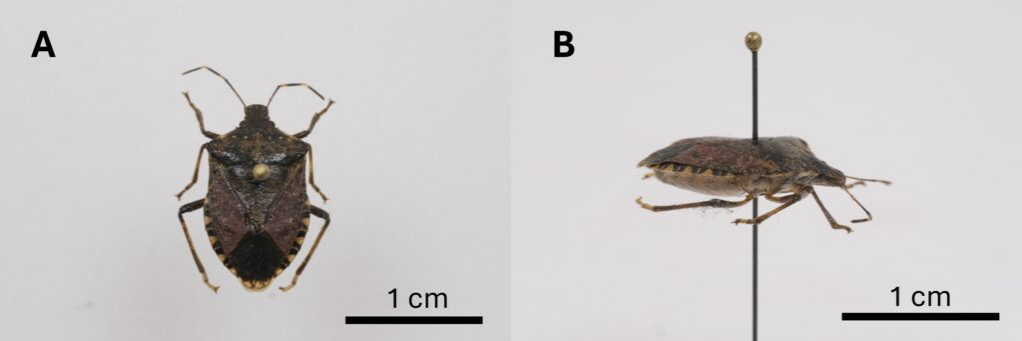
Pinned specimen of *Halyomorphahalys* (Stål, 1855) in dorsal view (**A**) and lateral view (**B**). Credit photo: Hugo Luttenschlager (ULiège).

**BELGIUM. Brussels-Capital.** *Ixelles* – 1 individual, 50.81254N 4.383688E, ULB Solbosch campus, 6.III.2023, dead individual found in ULB building, E. Garone leg.; 5 individuals, 50.814959N 4.383001E, ULB Solbosch campus, 28.VII.2023, collected with a sticky band containing an aggregation pheromone (Pherocon ^®^, Trécé Inc., Adair, OK, USA), L. Rossini leg. **Liège.** *Cointes* – 4 individuals, 50.623048N 5.556604E, 9.XI.2023 (1 individual; Fig. 2), 11.XI.2023 (2 individuals), 13.XI.2023 (1 individual), J. Carpentier leg.; *Waremme* – 3 individuals, 50.697688N 5.254619E, 15.VIII.2023 (1 individual), 09.IX.2023 (1 individual), 07.X.2023 (1 individual), F. Francis leg.; **Flemish Brabant**. *Hoeilaart* – 1 individual, 50.767747N 4.498898E, 30.VI.2023, collected near *Corilusavellana* (L.) with a sticky band containing an aggregation pheromone (Pherocon^®^, Trécé Inc., Adair, OK, USA), E. Garone leg.

The link of their records can be found here: http://doi.org/10.15468/kuxuek

## Discussion

In contrast to the methodologies of crowd-sourcing data and participatory sciences, the trapping and collection of *H.halys* specimens in Belgium remains relatively sparse, as highlighted by a mere three specimens recorded by Claerebout et al. (2018), 99 specimens recorded by Berteloot et al. (2024) and the 14 supplementary records from this study. Nevertheless, this limited dataset provides sufficient evidence to infer the successful establishment of *H.halys* within Belgium. The weather conditions in this area of the European continent are, in fact, favourable for the development of this species (Haye et al. 2014) even if the number of generations per year is still undefined. For example, the species has been assessed as close to two generations in Italy and south Europe (Costi et al. 2017, Rossini et al. 2023) and we expect to have at least one generation per year, based on the Belgian climatic conditions.

In this study, we had the opportunity to work with a large dataset of validated identifications made by insect specialists. The sampling effort accumulated by Belgian crowd-sourcing platforms (i.e. Observation.be Paquet et al. (2024), Vanreusel et al. (2024)) significantly supplements (96%) the dataset gathered by entomologists in the field (e.g. Berteloot et al. (2024)). This study demonstrates that validated data from citizen science are highly effective for understanding the distribution of an invasive species at a national scale (Chartois et al. 2021, Streito et al. 2021, Roy et al. 2024). In addition to validation by insect specialists, it is possible to verify specimens reported and photographed by users through automated validation methods, such as deep-learning approaches, based on convolutional neural networks (CNN) (e.g. in Tannous et al. (2023)). However, despite advances in image recognition methods in entomology, these methods still introduce inaccuracies into the dataset, as highlighted by studies indicating substantial rates of misclassification.

GBIF dataset showed a peak in sightings of *H.halys* that can be primarily attributed to its overwintering behaviour as already reported by Costi et al. (2017). This phenomenon of aggregation of the population inside living houses or buildings is generated by vibrational signals (Bedoya et al. 2020). It is worth noting, however, that the peak of the overwintering population is biased by the easier detectability of the specimens in the human living environments, leading to an increase of reports by urban or rural citizens in crowdsourcing platforms via smartphones.

Population genetic studies carried out on Belgian specimens (Berteloot et al. 2024) have suggested an expanding distribution of this species, especially in the northern regions of the country (i.e. Flanders). This region is featured by a high prevalence of apples, pears and soft fruits orchards, host plants that likely facilitates the proliferation of *H.halys*. The observed high genetic diversity of *H.halys* within Belgium (Berteloot et al. 2024) probably arises from multiple introductions endorsed by the global trade dynamics of the recent years (Valentin et al. 2017). Accordingly, the spatial distribution of *H.halys* should positively correlate with orchard density across Belgium (Hahn et al. 2017). The apparent disparity in occurrence of *H.halys* between Wallonia and Flanders could be explained by a significantly higher cultivation of orchards, as their apples and pears crop surfaces account respectively for 1640 ha and 14229 ha (i.e. ~ 10% vs. ~ 90%; Statbel (2022)), which represent preferred habitats for this species (Lee et al. 2013, Rice et al. 2014). However, this apparent gap could also stem from discrepancies in sampling efforts, particularly the under-representation of the French-speaking region in entomological documentation through participative sciences.

## Conclusion

In conclusion, we can say that the presence of this pest is currently homogeneous in the overall country and further experimentation devoted to assessing the potential damage induced on the different cultivations should be carried out. This is actually an important aspect to prevent higher outbreaks, as happened in different countries of Europe (i.e. Italy) (Maistrello et al. 2018), where the diffusion was slow for the first few years and then it increased to serious damaging thresholds.
